# Spiral Computed Tomographic Evaluation and Endodontic Management of a Mandibular Second Molar with Four Roots. A Case Report and Literature Review

**Published:** 2013-05-01

**Authors:** Aamir Rashid Purra, Mubashir Mushtaq, Irfan Robbani, Riyaz Farooq

**Affiliations:** 1 Department of Conservative Dentistry and Endodontics, Government Dental College and Hospital Srinagar, India; 2 Department of Radio diagnosis, Sher-i-Kashmir Institute of Medical Sciences, Kashmir, India

**Keywords:** Endodontic Treatment, Mandibular Second Molar, Spiral Computed Tomography

## Abstract

The vast majority of mandibular second molars have two roots with three root canals; however, variations in molar root canal anatomy are not uncommon. To the best of our knowledge, four rooted mandibular second molar with three mesial roots and one distal root has never been reported. Herein, we present the endodontic management of a four rooted mandibular second molar tooth, diagnosed with the assistance of spiral computed tomography (SCT) with a brief review of literature.

## 1. Introduction

Success of endodontic treatment relies on thorough chemomechanical preparation of the entire root canal system and its hermetic seal with an inert material. Various reasons have been cited in the literature for failure of endodontic treatment; one of the main reasons being the presence of undetected extra roots and canals (1). Therefore, it is mandatory for the clinician to have a thorough knowledge of the root canal anatomy and its variations (2). With the availability of such promising and exciting diagnostic tools as spiral computed tomography (SCT), new vistas have been opened in the non-invasive evaluation of dental morphology. Herein, we present SCT evaluation and endodontic management of a patient having four rooted mandibular second molar with three mesial roots and one distal root with a brief review of literature. To the best of our knowledge, such complex morphology has been rarely observed and reported.

## 2. Case Report

A 21-year-old male patient referred to our department with a chief complaint of severe pain in the lower right posterior teeth, during the last 5 days. The patient’s medical history was non-contributory. Dental history revealed presence of temporary fillings in the mandibular right first and second molars; these restorations were placed by a general dentist one month previously. On clinical examination, the right mandibular second molar was tender to percussion.

Digital radiographic examination (Schick technologies, NY, USA) of the involved tooth revealed the presence of coronal radiolucency involving the pulp; there was no evidence of any associated periapical radiolucency. Imaging also revealed the presence an extremely diverging distal root and a bulky mesial root, suggesting possibility of extra roots.

**Figure 1.**
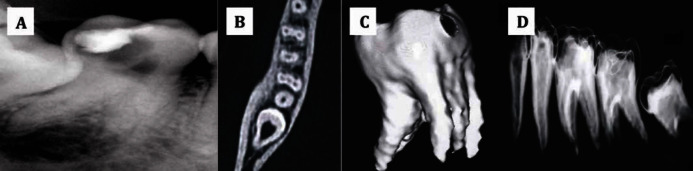
A) Pre-operative diagnostic radiograph; B) spiral CT scan showing the presence of three independent mesial canals; C) three dimensional reconstruction of the mandibular second molar with 3 mesial roots; D) spatial orientation of the mandibular second molar.

**Figure 2.**
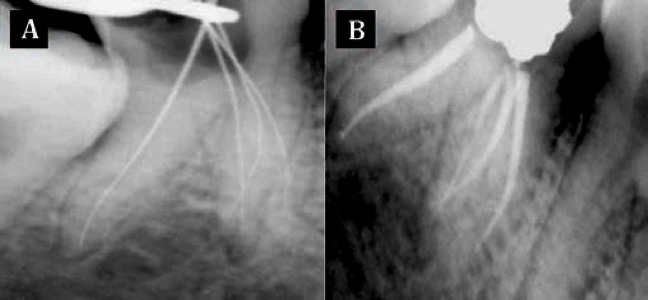
A) Working length confirmatory radiograph; and B) immediate post-obturation radiograph

Informed consent was obtained and endodontic treatment was planned for the molar to relieve the patient’s pain. Under rubber dam isolation, the access cavity was prepared and two canals (one mesial and one distal) were located at the first appointment. The access cavity was sealed with a temporary restoration. To assess the complex root canal anatomy, SCT of the involved tooth was planned. SCT images were obtained at a resolution of 0.6 mm and using dedicated dental software, mandibular bone subtraction was done. Three dimensional images were then reconstructed, using multi-planar reconstruction and surface shaded display techniques. CT images clearly revealed presence of three independent mesial roots and one distal root (Figure 1). At the next appointment, the access cavity was refined to search for the presence of extra canals; three independent mesial canals and one distal canal were explored using a DG16 endodontic explorer. All the four canals were negotiated using K-flex files (Dentsply Maillefer, Ballaigues, Switzerland). The working length determination was done using Elements diagnostic apex locater (Sybron Endo, Sybron Dental, Anaheim, CA, USA) and confirmed radiographically. The canals were then prepared with ProTaper rotary instruments (Dentsply Maillefer, Ballaigues, Switzerland) using copious Glyde (Dentsply Maillefer, Ballaigues, Switzerland) as a lubricant during the preparation. Canal disinfection was performed using 2.5% sodium hypochlorite. After root canal cleaning and shaping, the canals were properly dried with absorbent paper points and obturated with gutta-percha and AH plus sealer (Dentsply-DeTrey, Switzerland) (Figure 2). The access cavity was sealed with a temporary restorative material and the patient was recalled for permanent restoration at a later date.

## 3. Discussion

Dental radiographic evaluation is a fundamental tool for endodontic diagnosis. Conventional intraoral periapical radiographs are routinely employed to evaluate the root canal anatomy, but they are not very useful in the assessment of complex root canal anatomies due to their inherent limitations. The main constraint with conventional/digitally captured radiographs is that the three dimensional anatomy is compressed into a two dimensional image (3); such limitations do not allow precise assessment of complex endodontic morphology.

Since its application in endodontics in 1990 (4), CT scanning has become very popular with clinicians in the past few years. The successful use of SCT in the assessment of complex endodontic cases has previously been reported (5-10). With the availability of SCT, thorough examination of the entire root canal system has now become possible as thin slices of dental roots and canal systems can be viewed. Furthermore, advanced dental soft-wares allow 3D reconstructions of images across a multitude of planes.

Morphologically, the mandibular second molar resembles the first molar, except that the roots are shorter, more curved and there are more anatomical variations (11). The mesial root has two canals in 64% of cases and in 92% of cases the distal root has only one independent canal. Variations associated with mandibular molars have been demonstrated in several studies (12-17). Manning evaluated the canal anatomy of 149 extracted mandibular second molars using clearing technique. It was found that 22% of the mandibular second molars had single roots, 76% had two roots and 2% had three roots (18). Another study reported two cases of three rooted mandibular second molars, with one mesial and two distal roots (19). Costa Rocha *et al.* studied the anatomy of 628 extracted mandibular first and second molars; their results showed that 84.1% had two separate roots, 15.9% had fused roots and 1.5% had three roots (20). A further study evaluating racial variations of the mandibular second molar showed that the incidence of three rooted mandibular second molars was 2.8% in Mongoloid patients, 1.8% patients of Negro origin and 1.7% in Caucasian patients (21). Pineda and Kuttler conducted retrospective investigation of 7275 root canals and found four root canals with four apical foramina in 3.8% of cases (22). A case report documenting a mandibular second molar with five canals has also been reported (23). The presence of four roots (three mesial roots and one distal root) in the mandibular second molar is a very rare anatomic variant. A few cases of four rooted mandibular first molars have been documented in literature (24, 25). Recently, Peiris and colleagues presented a case of extracted mandibular second molar with four roots (2 mesial roots and 2 distal roots) using clearing technique (26). Our patient had a complex mandibular second molar morphology. With SCT we could confidently depict the presence of very rare three independent mesial roots and one distal root; precise portrayal of anatomy would not have been possible with conventional two dimensional radiography systems.
